# English general practice in a period of change: a mixed-methods study of staff and patient perspectives

**DOI:** 10.3399/BJGPO.2025.0069

**Published:** 2026-03-11

**Authors:** Heather Gage, Alice Herron, Bridget Jones, Simon Bailey, Amanda Bates, Rebecca Cassidy, Catherine Marchand, Emily McKean, Karen Spilsbury, Suzanne H Richards, John Campbell, Rupa Chilvers, Peter Williams, Phelim Brady, Mark Joy, Simon de Lusignan, Stephen Peckham

**Affiliations:** 1 Surrey Health Economics Centre, Faculty of Health and Medical Sciences, University of Surrey, Guildford, UK; 2 Centre for Health Services Studies, University of Kent, Canterbury, UK; 3 Faculty of Medicine and Health, University of Leeds, Leeds, UK; 4 Medical School, University of Exeter, Exeter, UK; 5 Bold Sparks, Exeter, UK; 6 School of Mathematics and Physics, University of Surrey, Guildford, UK; 7 GP Teams Project Service Users Panel, Guildford, UK; 8 Royal College of General Practitioners Research and Surveillance Centre, Nuffield Department of Primary Care Health Sciences, University of Oxford, Oxford, UK

**Keywords:** general practice, surveys and questionnaires, online focus groups, virual focus groups, appointments and scheduling, access to primary care

## Abstract

**Background:**

The COVID-19 pandemic prompted widespread use of remote (telephone and online) communication in general practice in England, which exacerbated long-term pressures from staffing shortages. The public perceived problems with access.

**Aim:**

To explore patient and staff perspectives on changing processes in general practice.

**Design & setting:**

A mixed-methods study (patient survey and staff focus groups) in a sample of 22 general practices in England, varied by size, region, deprivation, and demography, was conducted in 2022.

**Method:**

An online survey was delivered by short message service (SMS) text to adult patients at 21 practices. Data from answers to an open-ended question about patients’ experiences were analysed using summative content and thematic analysis. Virtual focus groups conducted with four categories of staff (GPs, nurses, receptionists and administrators, and practice managers) covered teamworking, roles, patient interactions, adapting to change, and workload. Data were transcribed and analysed using framework and thematic methods. Themes common to patients and staff were identified.

**Results:**

Overall median survey response was 10.9% (interquartile range 9.7%–14.6%); 14 401 patient responders provided 10 348 comments, 51.2% were positive. Patient and staff perspectives overlapped in two areas. The first, ‘contact and communication’, encapsulated differing views around access. The second, ‘non-clinical roles and patient care’, concerned the allocation of appointments and the roles of receptionists. Patients reported barriers to getting timely appointments with their chosen professional while staff were seeking ways to manage the volume of communications. Use of non-clinical staff to triage appointment requests was unpopular with patients and receptionists felt clinically unqualified.

**Conclusion:**

Effective methods are needed to improve patient communication with practices and access. Receptionists require recognition and training for their pivotal role.

## How this fits in

Pressures in general practices in England owing to rising demand and staff shortages were exacerbated by the COVID-19 pandemic. This investigation of patient and staff experiences in a varied sample of general practices in 2022 found two key areas of overlapping concern around patient access and receptionists’ roles. Solutions are required to persisting problems around access: patients report problems contacting practices and getting appointments to meet their needs, while staff report being overwhelmed by the ‘explosion’ of electronic communication. Non-clinical staff are increasingly taking on triage roles, which is unpopular with patients, and receptionists feel unqualified for this task.

## Introduction

Access and the debate about providing care remotely are central to concerns about the sustainability of general practice in England.^1^ For many years before the COVID-19 pandemic, demographic, systemic, and societal pressures that were threatening the patient experience had been building.^2^ Demand had increased owing to an ageing population with complex health and care challenges. Rising patient expectations and the transfer of tasks from hospitals to community settings added to the workload in general practice, and staff recruitment and retention had become serious problems.^3,4^ The situation was exacerbated in March 2020 when the UK government, in response to COVID-19, triggered abrupt changes in service delivery by instructing general practices to conduct consultations remotely (telephone and online) whenever possible.^5–7^ New systems resulted in the emergence of ‘modern’ receptionists. The traditional role of receptionists of issuing doctor appointments and liaising on home visits has transitioned into managing multidisciplinary options and flagging up alternatives to patients, including by remote contact. Receptionists may also be charged with triaging patient requests for consultations with the aim of optimising resources.^8^ The public, however, perceived serious problems with access.^9–11^ Concerns were raised about the safety of care delivered remotely and confidence in consultations declined.^6,12–15^

Various initiatives to address the workforce crisis (for example, technology, extended use of mid-level practitioners, and shared funding for additional roles such as physiotherapists and physicians’ associates) required practices to constantly reassess and adjust ways of working. Patients faced uncertainties around accessing care, and staff sought a manageable workload.^16^ This study draws on data from two elements of a larger study exploring patient and staff perspectives on changing processes in general practice (National Institute for Health and Care Research [NIHR] Health and Social Care Delivery Research Programme, project number: 17/08/34). Themes common to both patients and staff were triangulated to inform practices, policies, and future research.

## Method

### Design and setting

A sample of general practices was recruited. Information was distributed to practices across England by NIHR Clinical Research Network (now Research Delivery Network) regional offices. Expressions of interest were received from 72 practices. Data on these practices were compiled in a spreadsheet (region, number of registered patients for practice size, age distribution of patients, deprivation (Index of Multiple Deprivation) decile, urban or rural, and workforce characteristics), then 25 were purposively selected to provide maximum variability and invited to participate. Of these, 22 agreed. Practices were each reimbursed £500 on completion of data collection.

### Data and analysis

Data comprised patients’ responses to an open-ended question (within a larger survey) and staff views expressed in focus groups. Data collection, preparation, and analyses were carried out independently for the patients’ responses and focus groups, before examination of findings for overlapping themes. Data were anonymised. A service-user panel of 12 members (patients and public) worked with the research team on refining the data collection instruments, coding framework, and the interpretation of findings.

#### Patient survey

The patient survey was set up in Qualtrics. Between February and May 2022 study information and survey links were distributed by practices to registered adult patients who had signed up to receive text messages. The survey comprised 22 structured questions about access and experience of the practice, and brief background information about the responder (age, gender, last use of practice, and employment status). An optional open-ended question stated: *‘The space below is for a short comment or notes you would like to add to describe your experiences of the practice, such as, for example: — helpful, interested, friendly, understanding, kind; — unhelpful, not interested, irritable, always too busy*’ (maximum 300 words) (see Supplementary Information S1).

One practice did not use text messaging to communicate with patients and did not participate. Responses from the remaining practices on the survey platform were exported to Microsoft Word files. Summative content analysis was used to analyse responses to the open-ended question.^17,18^ Two researchers (AH and BJ) analysed the data using annotation and colour-coding on paper copy. They discussed the generation of codes and definitions, comparing notes and agreeing criteria. The approach was discussed frequently, including with a third researcher (HG) as analysis progressed. Initial reading assessed length, detail, and variability of content. Patient comments were relatively simplistic in terms of length and use of language, so coding was semantic, attributing straightforward definition to unambiguous statements. Individual data were not sufficiently in-depth or comprehensive to facilitate an interpretive approach. Codes were drawn from the data of five practices and refined so that comments could be rated as positive or negative. Data from the remaining practices were then coded. Where individual responses covered multiple comments, each comment was coded and rated separately. A framework reflecting different categories of issues (or potential themes) was generated and this was reviewed and refined. Positive, negative, and mixed (positive and negative on the same issue) codes were counted across all data, and by category, and for individual practices, and proportions calculated. The categories were described in narrative form. Patient Participation Groups at participating practices were invited to review and comment on findings for their practice.

#### Focus groups

Focus groups for staff were convened between December 2021 and March 2022. Separate discussions were conducted for four staff categories: GPs; nurses; administrative and reception staff; and practice managers. Owing to the ongoing pandemic, groups were conducted virtually on Microsoft Teams. Full information was distributed to practices with a request to find volunteers among staff. Informed consent was provided before participation. Discussions lasted a maximum of 90 minutes and were audio-recorded and transcribed verbatim; synchronous ‘chat’ comments were retrieved. Topics covered included the following: teamworking; interactions with colleagues and patients; changing roles; workload; and job satisfaction. Group discussion was the preferred method of data collection because it enabled participants to explore and clarify views through the group process.^19^ Basing groups around key categories of staff provided a degree of homogeneity and equality.^20^

Focus groups, data processing, and framework analysis were undertaken by SB, AB, RCa, CM, EM, and KS. Data were coded using a framework developed by the research team and bounded by the research aims. Additional inductive thematic analysis was carried out within the framework by BJ and AH. Transcripts were annotated by hand using an iterative and reflexive process of semantic coding, clustering, and attributing themes.^21^ Further details can be found in Supplementary Information S2.

#### Synthesis

Findings from separate analyses of patient and staff data were considered in parallel, looking for overlapping themes. Data sources were triangulated to provide a more comprehensive understanding of common topics from patients’ and staff perspectives.

## Results

### Practice characteristics

The sample of 22 practices was largely representative of the national distribution of practices by size. Defining features of some practices included high deprivation and high proportions of older or younger (student) patients (Table 1). Practice staff numbers ranged from 7 to 109; most had a core team of GPs, nurses, administrative and reception staff, and managers; one practice was nurse-led with locum GPs. Two of the smallest practices had opted not to join other local practices in a primary care network (PCN), which would have given access to some additional shared health professional resources; five practices were part of a super practice comprising an entire PCN. In the most recent Care Quality Commission inspections, 20 practices had scored good and two were deemed outstanding; none were reported as requiring improvement or inadequate. In the 2021 national General Practice Patient Survey, the proportion of patients reporting a very or fairly good experience of their practice ranged from 61%–100%.

**Table 1.** Characteristics of the 22 general practices recruited to the panel, by practice size

Practice size^a^	*n*	Region of England, *n*			Setting, *n*			National deprivation quintile, *n*					Ethnicity, *n*	Percentage of patients aged ≥65 years, *n*				PMS or APMS contract (versus GMS), *n* ^b^	Prior research activity, *n*
		North	Middle	South	Urban or sub-urban	Inner city	Rural	1 and 2 (least)	3 and 4	5 and 6	7 and 8	9 and 10 (most)	>10% of patients non-White	<15%	15.0%–19.9%	20.0%–24.9%	≥25%		
<2500	3	3	0	0	1	0	2	0	2	0	1	0	0	0	0	2	1	1	0
2500–4999	0	0	0	0	0	0	0	0	0	0	0	0	0	0	0	0	0	0	0
5000–7499	5	2	1	2	3	1	1	1	1	1	0	2	3	1	1	1	2	3	3
7500–9999	6	4	1	1	4	2	0	1	1	2	1	1	0	1	5	0	0	4	1
10 000–14 999	2	1	0	1	2	0	0	1	0	0	0	1	0	0	1	0	1	1	2
≥15 000	6	2	2	2	4	1	1	1	1	2	2	0	0	3	2	1	0	1	0
Total	22	12	4	6	14	4	4	4	5	5	4	4	3	5	9	4	4	10	6

^a^National mean practice size in May 2022 = 9522 registered patients.^47^ ^b^The General Medical Services contract is the standard contract between practices and NHS England. Some practices voluntarily agree to a local contract, Personal Medical Services, or Alternative Personal Medical Services. APMS = Alternative Provider Medical Services. GMS = General Medical Services. PMS = Personal Medical Services.

### Patient survey

A total of 14 401 responses were received. The overall median response rate (from text messages delivered) was 10.9% (mean 13.4%, interquartile range 9.7%–14.6%) (Table 2). Most responses were from women (*n* = 8866 [61.6%]; *n* = 5369 [37.3%] men; *n* = 36 [0.2%] other gender identity; and *n* = 130 [0.9%] missing); 13 195 (91.6%) identified as White British. Around one-third were in full-time employment (*n* = 5078, 35.3%) and 4225 (29.3%) were aged ≥65 years; nearly two-thirds had been in contact with the practice in the previous 6 months (*n* = 9284, 64.5%). Reporting at practice level of a good experience varied considerably, ranging from 42.9%–98.3% (Table 2).

**Table 2.** Patient survey distribution, response rates, and experiences of access across 21 practices

Category	Median	Mean	SD	Minimum	Maximum	25th percentile	75th percentile
**Survey distribution and response rate across 21 practices**							
Percentage of patients aged ≥18 years on practice list as willing to receive text messages from practice	87.9	84.4	14.4	32.5	99.4	80.1	91.7
Percentage of text messages sent that were delivered	92.0	91.5	5.3	76.6	100.0	90.1	94.1
Response rate as percentage of text messages delivered	10.9	13.4	6.9	3.8	32.5	9.7	14.6
**Patient reported experiences across 21 practices**							
Percentage reporting fairly or very easy to get appointment^a^	47.8	48.5	24.7	16.3	98.0	29.4	62.1
Percentage reporting fairly or very easy to get through on the phone^a^	66.2	60.7	26.5	15.6	99.4	35.2	82.0
Percentage reporting fairly or very good experience of the practice^a^	74.0	71.0	17.8	42.9	98.3	55.8	84.3
Percentage thinking staff at their practice definitely or probably work well as a team	88.0	83.7	12.7	57.3	99.4	74.4	93.5

^a^Items taken from the annual national General Practice Patient Survey. SD = standard deviation.

Some participants did not answer the open-ended question; others offered several comments. Overall, 8408 (58.4%) participants provided a total of 10 348 comments (average 1.23 comments per person). The proportion of participants providing any comment was relatively even across practices (mean 58.8%, standard deviation [SD] 4.5%, median 59.8%, range 46.1%–66.5%). The lowest proportions were in the two most deprived practices. Those most likely to provide comments were women (61.4% versus 53.1% of others); people aged ≥65 years (68.2% versus 52.6% in younger groups); people without parenting responsibilities (60.1% versus 50.1% of parents or guardians of children aged <16 years); carers of others with long-term physical or mental health conditions, disability, or old age problems (64.0% versus 56.7% without); and people not in full-time employment (62.7% versus 50.6% of those working full-time) (χ^2^ *P*<0.001 for each comparison).

Considering all 10 348 comments, just over one-half (51.2%) were positive, about two-fifths (41.8%) were negative, and the rest were mixed, with considerable variability among practices (Table 3). The highest proportions of positive comments (85.8%, 98.1%, and 98.8%) were from the three smallest practices, each with <2000 patients; these practices also had the highest response rates (32.5%, 27.8%, and 16.7%). Practices with the largest proportions of negative comments were in urban or city areas with highest deprivation and ethnic populations. Older people offered significantly more positive comments (*n* = 8280 comments, Spearman rho -0.015, *P*<0.001), while parents, carers, and people in full-time employment offered more negative ones (all Mann–Whitney U *P*<0.001).

**Table 3.** Analysis of 10 348 comments from 8408 patients in 21 practices participating in the patient survey

Category		Mean, %^a^	SD	Median, %^a^	Minimum, %^a^	Maximum, %^a^
Number of comments per practice		492.8	284.9	405	148	1202
Proportion of comments per practice that were:	Positive	51.2	23.8	49.9	19.1	98.8
	Negative	41.8	22.6	40.6	1.2	74.9
	Mixed positive and negative	7.0	3.6	7.0	0.0	12.4
**Proportion of all comments in a practice that were regarding**						
Theme 1: Contact and access (*n* = 3256 comments, 31.5%)		28.8	11.3	28.7	8.8	47.2
Proportion of theme 1 (contact and access) comments that were:	Positive	22.5	29.7	10.3	1.3	96.9
	Negative	74.8	30.1	87.3	3.1	97.6
	Mixed positive and negative	2.8	2.4	2.2	0.0	8.3
Theme 2: Attitude, approach, interaction (*n* = 4761 comments, 46.0%)		49.4	12.6	47.0	33.4	78.2
Proportion of theme 2 (attitude, approach, interaction) comments that were:	Positive	74.3	16.4	75.2	40.7	99.5
	Negative	16.8	12.1	14.8	0.5	45.1
	Mixed positive and negative	8.9	5.6	8.4	0.0	22.0
Theme 3: Healthcare processes, provision (*n* = 1925 comments, 18.6%)		18.0	4.5	17.5	11.9	28.7
Proportion of theme 3 (healthcare processes, provision) comments that were:	Positive	49.4	25.3	53.6	9.6	100
	Negative	38.9	24.7	33.1	0.0	84.6
	Mixed positive and negative	11.7	6.8	11.2	0.0	27.2
Theme 4: Expressions of failure (*n* = 232 comments, 2.2%)		2.1	1.6	1.6	0.0	5.1
Theme 5: Extreme adverse situations (*n* = 174 comments, 1.7%)		1.7	0.9	1.4	0.0	3.6

^a^Unless otherwise stated.

**Figure 1.**
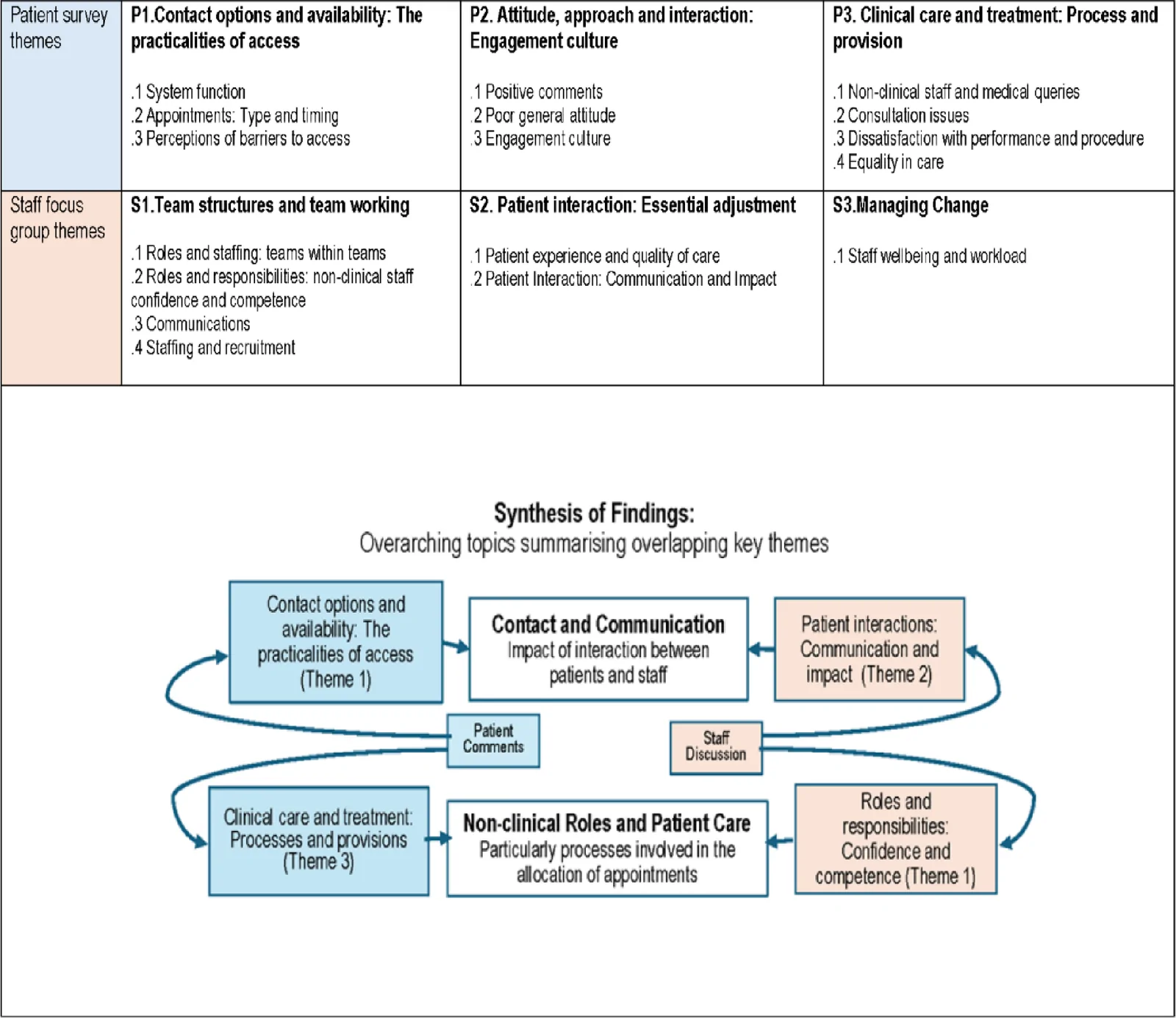
Themes from the patient survey and staff focus groups and synthesis of findings

Five themes were attributed to the data. There was variation among practices in the distribution of comments by theme (Table 3). Theme 1 (31.5% of all comments) covered contact with and access to the practice; across all practices, this theme had the highest proportion of negative comments. Theme 2 (46.0%) related to non-medical aspects, specifically staff attitudes and engagement culture; around three-quarters (74.3%) of these comments were positive remarks about staff. Theme 3 (18.6%) related to the processes and provision of clinical care and issues around consultations. The role of non-clinical reception staff undertaking triage to decide what health professional and type of appointment could be offered surfaced as an issue of widespread concern in this theme. The final two themes (<4%) captured grievances against the practice: expressions of failure where responders were considering leaving the practice or going elsewhere (for example, privately) because they could not get the care they wanted in a timely manner, and extreme accounts of misdiagnosis or wrong treatments. Themes 4 and 5 were combined with theme 3 in a revised three-theme framework (Figure 1).

### Staff views

A total of 38 staff volunteered for focus groups from 18 practices. Nine focus groups took place: two with nurses (*n* = 7); two with administrative and reception staff (*n* = 11); two with practice managers (*n* = 12); and three with GPs (*n* = 8). Groups were small owing to challenges of staff availability and scheduling.

The initial coding framework (see Supplementary Table S1) helped define three themes (Figure 1). The first related to team structures and teamworking covering roles and responsibilities, professional boundaries, and staff shortages. Teams within teams were identified by nursing and administrative and reception staff; participants from smaller practices and GPs and managers tended to identify with the whole practice. Issues were raised around responsibility for patient triage in most groups. Changes in colleague communication methods since the pandemic were described. WhatsApp groups were widely used and useful although issues around blurring of formal and informal messaging and the overwhelming amount of communication generated were expressed. The importance of face-to-face interactions and value of daily ‘huddles’ or coffee breaks with colleagues was acknowledged.

The second theme focused on the interface with patients and managing the ‘explosion’ of electronic communication after the pandemic. Staff recognised patients’ frustrations with access and expressed concerns that patient care would be jeopardised by increasing workloads. The third theme related to maintaining staff wellbeing in the changing, pressured work environment. Staff discussed wellbeing measures at their practices, including group yoga, lunchtime walks, and staff parties.

### Synthesis

Triangulating data from the patient surveys and staff focus groups identified two overlapping themes. The first, ‘contact and communication’, encapsulated different perspectives on accessing general practice (patient theme 1 and staff theme 2). The second, ‘non-clinical roles and patient care’, derived from feedback from both groups on the processes involved in the allocation of appointments (patient theme 3) and the roles and responsibilities of non-clinical staff (staff theme 1; Figure 1). These overlapping themes are presented below, with supporting comments in Tables 4 and 5.

**Table 4.** Patient and staff perspectives on overlapping theme 1: contact and communication

Perspective	Quote	Comments
Patient theme 1: Contact options and availability: the practicalities of access	4.1	‘*Dissatisfied with the telephone appointments last week on* [telephone] *for 4 hours and 15 minutes before l got through to reception and then told no telephone appointments left it's really bad after being on the phone for so long and told to ring again tomorrow.*’ (P1A p38) ‘*... the thought of trying to battle through the system to get an appointment means I will just put up with any ailments*.’ (P10 p2)
	4.2	*‘It takes far too long to make an appointment on the phone. The appointments are too far in the future ... econsult form invariably tells me to go to A and E when this is not necessary. I think the system is broken.’* (P14 p11)
	4.3	*‘It is very frustrating that if I want to book an appointment that isn’t urgent, I can’t get an appointment for weeks. So I end up doing an on the day urgent appointment, which makes me feel guilty.’* (P12 p5)
	4.4	*‘Very difficult to get a face-to-face appointment when necessary. It took 2 months ... from initial econsult, then a phone call appointment before the doctor agreed to see me. A recent phone appointment occurred out of the guide time so I missed it. No apparent understanding that patients might be working ... trying to keep well ... do not have all day to wait for a phone call or text.’* (P10 p27)
	4.5	‘*The website content is convoluted, cluttered, repetitious and overlapping. Not user friendly ... I often have to ring ... for clarification after sifting through the website ...* [Once] *I was given very misguided advice/information ... a result of lack of understanding on the reception part ... more phone calls ... ended up with an appointment with the surgery that should have been with* [an] *optometrist. So much wasted time and resources.’* (P7 p8)
	4.6	*‘Trying to do anything online is complex — website sends you down rabbit holes and round in circles. Difficult to get through on the phone.’* (P10 p20)
	4.7	*‘...* [askmyGP] *massively improved the experience of seeking help ... ”opening hours“ for online services* [have been] *heavily reduced ... varying day by day with no guarantee that it will be open ... they ”reach capacity” for online queries early in the day and then it’s back to queuing on the phone for 30 minutes plus to speak to reception, which may be completely unnecessary.’* (P9 p14)
	4.8	*‘It is impossible to get through to talk to anyone. The response time can be as long as 30 people before me. The Econsult is no longer available. I have asked for appointments with a doctor for both myself and my husband ... Cancer patient, 3 heart attacks, Covid ... A physician’s assistant responds to my requests for advice from a doctor and promises prescriptions and responses from specialist clinicians which never arrive or arrive 3–4 weeks later. I am not happy with the service and would move to another practice but have heard similar stories about alternatives.’* (P1A p49)
	4.9	*‘I only phone when I need to speak to a doctor, and I find they* [reception] *take control and tell you you can’t, very unhappy you can’t see a doctor!!!!!!!!’* (P16 p11)
	4.10	*‘... you first have to negotiate the receptionist ... they are the brick wall you have to negotiate. I’m told you need to give them the information to direct you to the best person. The best person is MY own doctor who would know my needs and not see a different person every time!’* (P10 p42)
	4.11	‘*Too hard to speak with actual doctors. Telephone receptionists asking too personal questions on why you are calling and then being unhelpful if you would rather not say for personal reasons.’* (P10 p27)
Staff theme 2: Patient interactions: communication and impact	4.12	*‘It’s not that we’re preferring it ... They don’t have to come here. And some of them think they can get things dealt with on the phone that can’t be dealt with.*’ (GP3/P1 L199-204)
	4.13	*‘For us, e-consults have just exploded, so we’ve now taken two of the receptionists totally off reception and they’re now care coordinators, and they deal with all the e-consults.’* (A1 /P4 L390-391)
	4.14	*‘... at first, it was brilliant, obviously, through COVID and everything. We’ve found it’s getting more and more difficult, and we’re just overrun at the minute.’* (A1/P1 L365–366)
	4.15	*‘We’d hit capacity by ten past nine ... it takes three of us* [admin] *just to manage that ... We don’t distribute appointments. Everything goes through ... askmyGP ... Even when it’s turned off, they’re ringing up ... they want to be dealt with today ... we think now they’re using us as a first port of call rather than a chemist, ‘cos they can just send a picture … ‘* (A1/P1 L365-378)
	4.16	*‘... two of the receptionists totally off reception and they’re now care coordinators ... so signpost patients elsewhere rather than the GP practice ... But the phones are still massively busy.’* (A1/P4 L390-394)
	4.17	‘*... there were just so many unnecessary consultations that were face to face previously that were basically a patient want rather than a patient need, and we can filter those out in far less time now and manage that in a much more sort of proactive way.’* (GP2/P8 L318–320)
	4.18	*‘... people who are really, you know, IT savvy ... They don’t want to put their information on a website where anyone can see it. They don’t want to tell the receptionist what the problem is.* [Citing a recent call] *... “I am not telling a doctor what is wrong with me on the telephone. I want an appointment. I want my bum on a seat.”’* (PM1/P5 L215-223)
	4.19	*‘... the majority of the phone calls after we’ve turned off, no matter what it is, we find that people have been a lot less patient ... — they want dealing with now.’* (A1/P1 L675-680)
	4.20	*‘... we put it in, like many others, quite quickly because of covid and it worked brilliantly ... trying to get out of the system ... is now incredibly hard. We’ve spent weeks and weeks trying to put a new appointment system in place, and changing over e-consult to now be only same-day assessment ... to bring back contacting the surgery directly, ‘cos now we’re getting the face to face and telephones back at the moment.’* (A1/P2 L418-428)
	4.21	*‘... a year and a half training patients how to use e-consult, which was difficult, and now it’s going to take that long again to train them to the new way we want ... patients are frustrated and finding it extremely confusing, and actually so are we. So, what was brilliant for one point in time has now become a bit of a conflict in our surgery.’* (A1/P2 L418-428)

Patient theme abbreviations: P = practice number; and p = page number. Staff theme abbreviations: as 2–3 groups were provided for each staff category, numerals beside the following three abbreviations indicate the focus group number for that staff category: A = administrative and reception staff; N = nurse; and PM = practice manager. Remaining staff theme abbreviations: L = line number in transcript. P = participant.

**Table 5.** Patient and staff perspectives on overlapping theme 2: non-clinical roles and patient care

Perspective	Quote	Comments
Patient theme 3: Clinical care and treatment: process and provision	5.1	*‘The receptionist seem to think they are the doctor and want to know why I need to see or want to speak to the doctor even if the doctor has asked me to call back ... ’* (P16 p6)
	5.2	‘*Very much dislike system of having to request a call from GP by explaining problem to receptionist. They are not qualified to triage and it is another hurdle to actually seeing the doctor. First you have to call, then get call back, then arrange an appointment.’* (P8 p6)
	5.3	*‘I didn’t feel comfortable* [about] *questions asked by receptionist who is not in my opinion medically competent to judge if I needed a face to face consultation or call back by GP.’* (P16 p7)
	5.4	*‘Receptionists often provide medical ”advice” and judgements (as they ask the reason for appointments) that they are no way qualified to give.*’ (P16 p27)
	5.5	*‘... you don't want to have to speak to the doctor about your problem with your colleagues listening. Also I don't want to talk to the receptionist about my problems. All these hurdles have made me delay booking an appointment about something I need to see the doctor about.’* (P8 p6)
Staff theme 1: Roles and responsibilities: confidence and competence	5.6	*‘... GPs don’t make a lot of decisions ... The only thing they decide is if they’re bringing a patient in face to face ... where the appointments go and how they get made is all left up to the admin and the one receptionist ...* [when available] *appointments are finished, the e-consult slots are full, but people are still ringing ... it’s left up to the admin team to decide what’s urgent enough to go on the on-call list.’* (A1/P7 637-642)
	5.7	*‘... It gets very difficult to feel like you’re doing what you’ve signed up for here, ‘cos sometimes you feel like you’re a brick wall before the GP and not necessarily the door into the GP. I’ve had patients say to me on the phone, “Why am I being triaged by you to see the doctor when all I want to do is see the doctor?” And I want to say to them, “I don’t want to do it, I don’t want to do it, but this is just how it works.”’* (A1/P7 650-654)
	5.8	‘*... we have a bit of a turnover in reception, but I would have thought it would be greater for the amount — they get so little benefit in comparison with the amount of hassle and abuse ... ‘* (PM1/P8 L845–847)
	5.9	*‘... nurse practitioners used to do our triage before covid hit and askmyGP came onto the scene ... far better, to be honest, for patients ... askmyGP has a receptionist that’s in charge of directing all requests ... And that can cause a muddle sometimes because they’re unsure who to direct things to, whereas when you had a practitioner doing your triage, it was directed far more effectively.* [Facilitator: And do you think that will go back ... ?] *Well, a couple of the GPs would prefer to, but then others wouldn’t, and so it depends really on who your GP is as to whatever. So, at the minute, no, it isn’t going to go back.’* (N1/P5 L692-697)
	5.10	*‘They* [non-clinical staff] *are starting to read the messages and the information that’s given to them more, which has improved things, and I think calmed the partners down a bit as well. ‘Cos the duty doctor always leaves their door open and listens to what’s going on, and then they come to me saying, “How many questions did that person ask today? Why don’t they know this? And why don’t they know that?” they’re stressing everybody out.’* (PM2/P1 L421-425)
	5.11	*’... we started like an online consult thing, that then the admin team deal with rather than reception. And the communication between the admin team and the GPs, it’s not great, because we’re having to work in so many different ways depending on which GP it is. Some of them want things doing one way. Some of them want things doing another way, which then can cause friction back to reception, because if reception have taken a phone call that’s been passed on to us for a GP but it’s not got certain information that one doctor would want and one wouldn’t ... ‘* (A1/P1 L270-278)

Patient theme abbreviations: P = practice number; and p = page number. Staff theme abbreviations: as 2–3 groups were provided for each staff category, numerals beside the following three abbreviations indicate the focus group number for that staff category: A = administrative and reception staff; N = nurse; and PM = practice manager. Remaining staff theme abbreviations: L = line number in transcript. P = participant.

### Overlapping theme 1: contact and communication

Patients’ descriptions covered functional aspects of access via telephone or online as well as availability of timely, suitable appointments. While some responders liked the convenience of telephone consultations, frustration was reported owing to queuing, being cut off, and too few same-day appointments that were all allocated soon after lines opened. The time, cost, and stress of making repeated calls deterred some participants who said they lived with problems rather than *‘battle through’* to make an appointment (quote 4.1). Patients from some practices took issue with time frames for non-urgent appointments that could involve a wait of 3–6 weeks (quote 4.2). Participants admitted to exaggerating the urgency of a problem to avoid waiting (quote 4.3). Getting an appointment was a particular issue for people in full-time employment who found it difficult to make or take personal calls during working hours. Frustrations were expressed when a telephone appointment with a doctor was missed because it was not made at the pre-arranged time (quote 4.4).

Comments highlighted individual differences in the acceptability of online engagement. Some criticised poorly designed websites that hindered access (quotes 4.5 and 4.6), while others who found online systems convenient were annoyed when the service was reduced (quote 4.7). Practice policy of offering appointments with an alternative practitioner when consultation with a GP was requested was an issue for many (quote 4.8). Many regarded receptionist and care navigator roles as a barrier to access *(*quotes 4.9, 4.10, and 4.11).

Staff described telephone or the online ‘askmyGP’ service as core ways for patients to contact practices; many consultations were also remote, by telephone or video link. While online communication with patients was described as largely successful and necessary, accounts revealed challenges from the process, technology, and workload, including task duplication, for example, when inadequate telephone consultations required face-to-face appointments (quote 4.12). Staff reported that patients used online contact to the extent that *‘e-consults have just exploded’* (quote 4.13) and it was *‘getting more and more difficult, and we’re just overrun’* (quote 4.14). Even though online services were sometimes temporarily closed as practices became overwhelmed, the workload was still unmanageable, with insufficient slots for appointments (quote 4.15). Staff acknowledged that congestion was a barrier to patient access even when patients could be steered to alternative sources of support (quote 4.16).

The clash between patient requests to see a GP and practice policies of diverting, where appropriate, to non-medical colleagues, was described as negotiating *‘a patient want rather than a patient need’* (quote 4.17). Ability to triage incoming requests and steer patients away from the practice to other sources of support (such as pharmacists) was seen as an efficient way of handling high levels of demand. Staff were sympathetic, however, to patient resistance to the triage system that requires them to divulge personal information online or to reception staff on the telephone, or in the reception area where they could be overheard (quote 4.18)*.*

Staff recognised that the system of engaging with patients was fraught with hitches and expressed understanding of patients’ problems getting through on the telephone. Staff were overwhelmed and patients disappointed, frustrated, and less tolerant when they were unable to log on (quote 4.19). One practice was trying to reinstate direct contact while retaining online access (quote 4.20) but the process seemed to have rendered patients as objects, *‘to train them to the new way we want’* (quote 4.21).

### Overlapping theme 2: non-clinical roles and patient care

Patients’ concerns about the processes and provision of clinical care and treatment covered many issues, including: loss of face-to-face appointments with GPs; lack of confidence in telephone diagnoses; limited time in consultations; missed health checks; non-communication of test results; and lack of continuity with the same GP. Most prominent, however, was the role of non-clinical staff and especially being asked by receptionists for personal information. Receptionists were regarded as neither qualified nor trained for carrying out triage and deciding whether a problem should be referred to a doctor (quotes 5.1, 5.2, 5.3, and 5.4). Participants did not anticipate having to voice personal details when they telephoned practices, so they were not necessarily in a private place (quote 5.5).

Discussions with staff covered receptionists’ roles and responsibilities, and reflected patients’ concerns about triage by non-clinical staff. Issues were raised around unwanted change in responsibility, inadequate training, competence, low confidence, and abusive behaviour from frustrated patients (quotes 5.6 and 5.7). There was awareness that changes in responsibilities deviated from original job specifications and several indications that they had been imposed. While there seemed to be overall agreement that the *‘hassle and abuse’* (quote 5.8) non-clinical staff received from some patients was unacceptable, comments mirrored patients’ perspectives that patient-facing staff were a barrier to access. There was an impression of administrative and reception staff ‘in uproar’, but clinicians were also aware of the problems related to non-clinical triage, with indications of concern from some (quotes 5.9 and 5.10). The process could be hindered by poor internal communication (quote 5.11).

## Discussion

### Summary

The study recruited practices of varying sizes serving differing populations across England 2 years after the onset of the global pandemic triggered the swift adoption of remote communication and consultations with patients. While overall around half of all comments from patients were positive (higher in three small practices), key issues reported by patients were difficulties contacting practices and getting appointments at a time, and with the professional of their choice, and concerns about the gatekeeping function of receptionists and their role in patient triage.

The challenges faced by patients in contacting practices for appointments were acknowledged by staff, along with concerns about communication with patients. Staff described being overwhelmed by the volume of patient communications, online as well as by telephone, but did not offer solutions or effective strategies. Comments indicated that patients were not considered as active contributors in practice processes, but were seen as needing training in understanding and complying with new methods.

There were concerns about functional aspects of non-clinical staff roles in all staff focus groups. Patients were unhappy about non-clinical staff processing medical information, while receptionists expressed fears about their competence in dealing with clinical details. Just as patients were frustrated by changes imposed on them, reception staff were unhappy about changes to their roles, made without consultation and with minimal, or no, training.

### Strengths and limitations

This study uncovers and explores issues from the perspectives of service users and providers. However, it has some limitations. Data collection was carried out within 6 months of the end of the second national COVID-19 lockdown when practices were still in a phase of adjustment, and this may have hindered recruitment of practices. Despite this, the objective to include at least 20 varied practices was met.

Patient participation was limited to those with smartphones and sufficiently familiar with technology to engage with a survey link sent by text message. The option for requesting a paper copy received only one request. Therefore, people with lower incomes, disabilities, or poor literary skills are likely to have been excluded. The survey was available only in English, therefore language issues may have prevented some from participating. Targeted outreach within practices to enable digitally excluded or non-English speaking patients to express their views was not feasible because of continuing restrictions after the pandemic. Patient perspectives obtained through the survey are based on responses to only one optional question. This was a pragmatic decision recognising the need to keep the questionnaire brief and simple to encourage responses. Response rates were low and some bias was observed with women, older people, parents, carers, and those not in full-time employment more likely to respond. Participants might have disproportionately been people with strong opinions, especially problems and concerns with the service or their care; non-responders may be those with a neutral view, lack of familiarity with the practice, or too busy. We sought confirmation from Patient Participation Groups as to whether responses were representative of the practice situation. Although it is now a statutory requirement for practices to have an active Patient Participation Group, some practices had not reconvened their groups after suspending them during the COVID-19 pandemic; those groups that were active indicated the comments of participants were valid. Several also described ongoing changes in their practices to mitigate problems that the survey had identified.

The recruitment of practice staff to focus groups was challenging owing to the COVID-19 winter wave of illness. Four practices offered no volunteers; six practices participated only in one focus group. Holding focus groups with staff online provided flexibility but some groups had small numbers of participants because staff could not attend at the last minute. It was particularly difficult to recruit GPs. Finding a private space was a challenge for some participants, meaning that there were occasional interruptions. Despite these challenges, important insights, experiences, and opinions were shared.

### Comparison with existing literature

Improving access to general practice has been a policy priority for many years.^22^ Access is a multi-component concept^23^ and many different approaches have been trialled.^24,25^ Recent research shows that rules and processes intent on improving access may exacerbate problems, particularly continuity of care^26,27^ and finding privacy in remote consultations,^28^ both issues raised by our participants. Consistent with our finding of patient dissatisfaction with telephone access, the annual national General Practice Patient Survey reported only 50% of responders stating it was fairly or very easy to get through to someone on the phone at their practice in 2023, a fall from 81% in 2012.^29^ In line with our results, evidence from patients and staff show mixed views about online tools. While some perceive convenience benefits, others are concerned that some patients are disadvantaged and that problems may be missed or treatment delayed.^30^ Digital work also creates technostress, technosuffering, and relational strain among staff.^31^ A big data analysis, however, argues that an online general practice access model using digital and non-digital pathways with triage can effectively manage patient-initiated demand, remain sensitive to patient preferences, prioritise care, and optimise workforce use, although implementation needs to reflect local context and requires support.^32^

Tasking receptionists with triaging functions has been identified as both a potential safety issue and inefficient since inappropriate decisions can result in multiple appointments instead of one face-to-face encounter.^14^ Participants in our study confirmed this finding and the conclusions of another recent study in which receptionists described their role as ambiguous and inadequately understood and recognised, and highlighted inadequate training for the clinically relevant aspects of their revised jobs.^8^ Patient dissatisfaction when receptionists cannot provide an appointment at a convenient time or with a preferred GP is a long-standing problem^33^ and sometimes results in aggression towards receptionists.^34–36^ This was noted in some focus groups and points to the need for special training and workplace measures to protect the wellbeing of reception staff.^36^ Early ethnographic research identified the influence of personal judgement and power, and issues of variability, subjectivity, inconsistency, and potential inequality in the receptionist role,^37^ and this was also apparent in our findings. A recent systematic review also confirmed our analysis of the overlap between the views of receptionists and patients, namely that receptionists are anxious about clinically oriented tasks, such as triage, while patients lack trust in receptionists and view them as obstructive and unqualified.^38^

### Implications for research and practice

The pandemic accelerated changes across general practice that were already evolving. The shift from a demand-driven service designed to meet patients’ requirements and in-person appointments to one limited by capacity^14^ and offering remote access has created a complex process of service delivery that staff and patients in many practices are learning to work with. Service users in small practices (<2000 patients) were highly positive and reported minimal access problems suggesting a need to explore the effect of practice size and organisation. Others, for example, have suggested that staff working in micro-teams or ‘teamlets’ might improve communication with patients, working environments, coordination, and continuity of care.^39–42^ For over a decade practices have been encouraged to work at scale to the detriment of continuity of care and the patient experience.^43^ In addition, as part of NHS England’s plans for a digital transformation of the NHS, practices must now put processes in place to ensure equitable access for people who might otherwise have difficulty seeking or benefiting from care, such as those who cannot afford a smartphone, have no internet, are disabled, or have poor literary skills.^44^

The pivotal role of receptionists in enabling access for patients and acting as gatekeepers to GPs and other health professionals^45^ merits further research. The role is recognised as challenging with emotional impact (‘emotional labour’) resulting from engaging with conflicting priorities and providing empathy.^33^ It is also influenced by factors largely outside the control of receptionists such as workplace culture, processes and protocols, digital technologies, and limited health service capacity.^46^

Receptionists require support to navigate the challenges of their role, training, and a career path that recognises its centrality for effective service delivery.^8^ Establishing a professional code of practice for receptionists, as exists for clinicians, would help define role boundaries, provide a basis for shared understanding across all staff groups, and enable patients to feel more secure in the triage process. This could be underpinned by a recognised training programme that goes beyond in-practice induction to cover issues such as dealing with sensitive information and difficult patients. Further research on frontline relationships in primary care is required to inform this. Training for all staff roles is required to facilitate effective engagement with patients. Systems need to be both efficient and patient friendly. Involving patients in decision making around practice processes is important for taking account of their preferences, improving understanding of constraints practices face, and offering ownership of adjustments that are implemented. While Patient Participation Groups are intended for such involvement, a practice champion could coordinate activity and ensure effective group function.

## Data

The dataset relied on in this article is available from the corresponding author on reasonable request.
